# Phenotypic Characterization of High Carotenoid Tomato Mutants Generated by the Target-AID Base-Editing Technology

**DOI:** 10.3389/fpls.2022.848560

**Published:** 2022-07-07

**Authors:** Johan Hunziker, Keiji Nishida, Akihiko Kondo, Tohru Ariizumi, Hiroshi Ezura

**Affiliations:** ^1^Graduate School of Life and Environmental Sciences, University of Tsukuba, Tsukuba, Japan; ^2^Graduate School of Science, Technology and Innovation, Kobe University, Kobe, Japan; ^3^Faculty of Life and Environmental Sciences, University of Tsukuba, Tsukuba, Japan; ^4^Tsukuba Plant Innovation Research Center, University of Tsukuba, Tsukuba, Japan

**Keywords:** carotenoid accumulation, base editing, high-pigment, tomato, new breeding technology

## Abstract

Our previous study demonstrated that Target-AID which is the modified CRISPR/Cas9 system enabling base-editing is an efficient tool for targeting multiple genes. Three genes, *SlDDB1*, *SlDET1,* and *SlCYC-B,* responsible for carotenoid accumulation were targeted, and allelic variations were previously obtained by Target-AID. In this research, we characterized the effect of new alleles on plant growth and fruit development, as well as carotenoid accumulation, individually in segregating backcross populations or combined in null self-segregant lines. Only lines carrying homozygous substitutions in the three targeted genes and the segregating backcross population of individual mutations were characterized, resulting in the isolation of two allelic versions for *SlDDB1,* one associated with *SlDET1* and the last one with *SlCYC-B.* All edited lines showed variations in carotenoid accumulation, with an additive effect for each single mutation. These results suggest that Target-AID base-editing technology is an effective tool for creating new allelic variations in target genes to improve carotenoid accumulation in tomato.

## Introduction

Gene editing is a technology for modifying a target gene by removing, inserting, or substituting DNA. Base-editing (BE) technology has been developed with the aim of inducing local targeted nucleotide substitutions. The clustered regularly interspaced short palindromic repeats (CRISPR)/CRISPR-associated protein 9 (CRISPR/Cas9) system is currently the most popular gene editing tool due to its efficiency and application to living organisms (Hsu et al., 2014). CRISPR/Cas9 tends to form more indels than substitutions, inducing gene knockouts, which can be deleterious depending on the gene or the position within it. To improve the efficiency of substitution, a number of BE technologies have been developed (Komor et al., 2016; Nishida et al., 2016; Gaudelli et al., 2017; Anzalone et al., 2019).

Most BE technologies are based on an engineered Cas9 protein fused with a single strand DNA-specific cytidine deaminase (CDA; Komor et al., 2016; Nishida et al., 2016; Nishimasu et al., 2018). Target-AID base-editing technology, in which an *Arabidopsis thaliana*-optimized codon nickase Cas9 (nCas9) fused with a lamprey CDA is used, has been demonstrated as a powerful tool for precise BE for multiplexing applications in bacteria or plants (Banno et al., 2018; Shimatani et al., 2018; Hunziker et al., 2020).

Tomato is one of the most important commercial horticulture crops grown worldwide. Improvement of yield and quality in tomato is a major challenge for breeders, as fruit yield and quality are controlled by several genes acting either independently or in concert. Tomato fruit is known as a good source of lycopene, vitamin C, β-carotene, folate and potassium (Willcox et al., 2003). Carotenoids play an important role in human nutrition because of their pro-vitamin A activity. However, there are other significant health benefits that are attributed to carotenoids; these benefits are thought to be associated with their antioxidant activity as well as their anticancer potential. Several genes involved in the improvement of carotenoid accumulation have been identified in tomato during the last two decades: tomato DNA damage UV binding protein 1 (*SlDDB1*; Lieberman et al., 2004; Liu et al., 2004), de-etiolated1 (*SlDET1*; Mustilli et al., 1999) and lycopene-β-cyclase (*SlCYC-B*; Ronen et al., 2000) for lycopene accumulation, and *SlCYC-B* (Ronen et al., 2000; Hwang et al., 2016; Mohan et al., 2016), *SlCRTISO* (Isaacson et al., 2002) and *ORANGE* (Lu et al., 2006; Tzuri et al., 2015; Yazdani et al., 2019) for β-carotene accumulation.

Previously identified alleles of tomato involved in lycopene accumulation such as DNA damage UV binding protein 1 (*SlDDB1*) names *hp1* (Lieberman et al., 2004; Liu et al., 2004) and de-etiolated1 (*SlDET1*) named *hp2* (Mustilli et al., 1999) showed a significant impact on plant and fruit development, such as dwarfism and fruit development delays (Wang et al., 2019). Several agronomic studies have already shown the possibility of improving agronomic traits such as lycopene accumulation in tomato fruit. The combined effects of an allelic version of *hp* (*high pigment*) mutant family genes with the *Slog* mutant allele resulted in higher lycopene accumulation in fruits (De Araújo et al., 2002; Ventura Faria et al., 2003; Andrade et al., 2015). However, there have been no studies on carotenoid accumulation in the double mutant line *SlDDB1/SlDET1*, except for a single report on extreme dwarfism during photomorphogenesis in seedlings (Liu et al., 2004).

In this study, we analyzed new allelic versions in tomato mutants that were obtained by Target-AID base-editing technology in our previous study (Hunziker et al., 2020) for their impact on plant growth and fruit development. The three targeted genes, *SlDDB1*, *SlDET1* and *SlCYC-B*, play a role in carotenoid accumulation, especially lycopene, in tomato. Several studies have been conducted using the double mutants *hp1/og* and *hp2/*og (De Araújo et al., 2002; Long et al., 2006; Sestari et al., 2014); however, the use of the *SlDDB1*/*SlDET1* double mutant for carotenoid accumulation has not yet been reported in tomato fruits. Therefore, we used multiplexed targeted plants and targeted the *SlDDB1*, *SlDET1* and *SlCYC-B* genes from our previous study (Hunziker et al., 2020). This approach resulted in the generation of several lines showing an improvement in lycopene accumulation *via* the stacking of mutations. Moreover, these lines did not show strong deleterious effect compare to the WT such as extreme dwarfism of plant or strong delay in fruits maturation reported in previous study (Liu et al., 2004; Wang et al., 2019).

## Materials and Methods

### Plant Culture

Tomato (*Solanum lycopersicum*) cv. Micro-Tom Japan and the mutants generated in our previous study (Hunziker et al., 2020) were used in this study. T_3_ null segregant lines resulting from self-pollination of the lines #16-3_3, #43-2_2 and #43-2_6’ and BC_1_F_3_ lines resulting from a back cross between WT parents and the line #16-3_3 containing a single substitution in *SlDDB1* were used. Plants were grown in a culture chamber under an LED photosynthetic photon flux of 270 μmol.m^−2.^s^−1^ light (16 h/8 h) at a constant temperature of 25°C.

### PCR and Genotyping Analysis

For PCR amplification, the primers used in this study are listed in Supplementary Table S1. For Sanger sequencing, targets were amplified using KOD FX Neo (Takara, Japan) according to the manufacturer’s protocol, and the PCR product was directly purified using the FastGene Gel/PCR Extraction kit (Nippon Genetic, Japan).

For CAPS analysis of *SlDET1* and *SlCYC-B*, targets were amplified using KOD FX Neo enzyme (Takara, Japan) according to the manufacturer’s protocol with primers designed for Sanger sequencing. The PCR product was directly digested with the restriction enzymes AvaII and MscI (New England Biolabs, United States), respectively, for *SlDET1* and *SlCYC-B*, both enzymes cutting in the WT version. A 10x diluted PCR product was used as the template, and the reaction followed the manufacturer’s recommendations, with an incubation at 37°C for 1 h using an appropriate buffer concentration. dCAPS primers were designed for *SlDDB1* using the dCAPS Finder website;^1^a1 mismatch maximum was used for the generation of primers to create a HindIII restriction site in the WT allele of *SlDDB1*. The presence or absence and pattern of the mutations was confirmed in segregating BC_1_F_3_ plants using Sanger sequencing.

### Analysis of the Impact of a Mutation on Plant Phenotypes in T_3_ and BC_1_F_3_ Lines

To compare the impact of individual mutations on plant phenotypes, several agronomical traits were analyzed. Seedlings undergoing photomorphogenesis from different lines carrying mutations in *SlDDB1* and *SlDET1* were grown under the same light conditions as control plants under an LED photosynthetic photon flux of 270 μmol•m^−2.^s^−1^ light (16/8 h) or under total darkness for 7 days, at a constant temperature of 25°C. Seeds were treated with bleach for 15 min followed by 4 washes with autoclaved tap water and transferred to half-strength MS medium in a magenta box. The box was placed in the dark for 2 days and then under light for 5 days for the light experiment, or kept in the dark for 6 days followed by 1 day in the light. Hypocotyl length was measured using a digital caliper (Mitutoyo, Japan).

For plant and fruit size measurements, lateral shoots were removed to retain a single shoot with 3 trusses per plant, with a minimum of 5 fruits per plant. The impacts of each mutation on plant size were analyzed for 90 days post-germination, when plants were considered mature. For fruit measurements, fruit weight, equatorial size, and days from anthesis to the breaker stage were observed from the breaker stage to 12 days post-break to compare the impact of *hp* mutations and maturation on fruit development.

### Carotenoid and Chlorophyll Analysis

Carotenoid and chlorophyll analyses were performed as described below. For the carotenoid analysis, five red fruits per plant were harvested at 12 days post-break (DPB). Subsequently, the pericarps were frozen in liquid nitrogen and then ground to a fine powder by using a Multi-Bead Shocker (Yasui Kikai, Japan). For the chlorophyll content, five green fruits per plant were harvested at the green mature stage, corresponding to 28 DPA, and then treated as described above. The powder was stored at −80°C and used for different purposes. For T_3_ experimentation, 6 plants from each triple mutated line were analyzed and divided into 3 individual technical replicates. For the BC_1_F_3_ generation, 4 plants per line were used and divided into 3 technical replicates for each plant. To perform pigment extraction, 100 mg of the powder was used to extract chlorophyll, lycopene and β-carotene in accordance with the procedures described by Nagata and Yamashita (1992) and Anthon and Barrett (2007), with small modifications.

Chlorophyll extraction was performed using 1.5 ml of acetone-hexane (4:6 v/v) in a 5 ml tube that was then vortexed and sonicated for 20 s. All samples were kept on ice and protected from light to prevent degradation. The samples were briefly centrifuged. Then, 700 μl of the organic phase was transferred to a quartz cuvette inserted into a spectrophotometer (SpectraMax M2, Molecular Devices, USA). The absorbance was measured at 645 nm (A_645_) and 663 nm (A_663_). The contents of chlorophyll “a” and chlorophyll “b” were calculated using the following equations:


(1) $$\mathrm{Chlorophyll}{\;}^{‘}a^{’}\left(\mu g/\mathrm{gFW}\right)=\left(0.99{\mathrm{9A}}_{663}-0.0989A_{645}\right)\times 100$$

(2) $${\mathrm{Chlorophyll}}^{‘}b^{’}\left(\mu g/\mathrm{gFW}\right)=\left(-0.32{\mathrm{8A}}_{663}+1.77A_{645}\right)\times 100$$

For carotenoid extraction, the powdered sample was placed in 8 ml of a hexane-acetone-ethanol (2:1:1 v/v) solution, vortexed and sonicated for 20 s and then incubated in the dark for 10 min under agitation to complete the extraction. After 10 min, 1 ml of deionized water (DW) was added to the solution, which was then vortexed and incubated for 10 min in the dark without agitation to perform organic phase separation. Then, 700 μl of the organic phase was transferred to a quartz cuvette. Absorbance was measured at 444 nm (A_444_) and 503 nm (A_503_) by a spectrophotometer. The volume of the extraction solution (V) and sample weight (W) were recorded to adjust the following equations. The contents of lycopene and β-carotene were calculated as follows:


(3) $$\begin{array}{l} \mathrm{Lycopene}\;\left(\mu g/\mathrm{gFW}\right)=\left(6.95A_{503}-1.59A_{444}\right)\\ \times 0.55\times 537\times \left(V/W\right) \end{array}$$

(4) $$\begin{array}{l} \beta -\mathrm{carotene}\;\left(\mu g/\mathrm{gFW}\right)=\left(9.38A_{444}-6.71{\mathrm{0A}}_{503}\right)\\ \times 0.55\times 537\times \left(V/W\right) \end{array}$$

### Sugar and Protein Content Analysis

Sugar contents, including glucose (Glc), fructose (Fru) and sucrose (Suc), were analyzed using enzyme activity analysis. These metabolites were extracted from 15 mg fresh weight of powder using an ethanolic fractionation protocol with 1.5 ml screw-capped tubes (Hendriks et al., 2003). Following the extraction step, metabolites were quantified using enzyme activity assays and measuring the absorbance of NADPH produced by spectrophotometry using a plate reader spectrophotometer (SpectraMax M2, Molecular Devices, USA; Stitt et al., 1989; Gibon et al., 2002). Succinctly, 50 μl of the ethanolic fraction was added to 160 μl of enzyme analysis buffer containing G6PDH (Roche, Germany), and the sample was incubated at 37°C and observed at 340 nm until the absorbance value stabilized. After the plateau was reached, hexokinase (Roche, Germany), phosphoglucose isomerase (Roche, Germany) and invertase (Sigma, USA) were added to analyze the quantities of Glu, Fru and Glc, respectively. The delta of the absorbance was calculated between each analysis, and the quantity of sugar was expressed in μmol/g FW compared to a standard curve. Sucrose content were expressed in equivalent glucose.

Proteins were extracted from the remaining pellet, and 400 μl of 0.1 M NaOH was added. Samples were heated to 95°C for 30 min with vortexing every 5 min and then cooled at room temperature. The samples were briefly vortexed and centrifuged at 2,500 rpm using a shaking table. Then, 3 μl of supernatant or bovine serum albumin (BSA) was added to 180 μl of Bradford stock reagent (Wako, Japan) in a 96-well ELISA plate, and the absorbance was measured at 595 nm. The absorbance was compared to the standard curve, and the protein content was expressed in μg/gFW.

### Subcellular Localization of *SlDET1* Based on Loss of Function

Observations were made on the correlation of the effect of the loss of function with loss of localization of *SlDET1* resulting from NLS mutation using transient expression of WT and mutated versions of the protein fused to YFP. WT and mutant RNA were extracted from leaves using an RNA Plant Mini Kit (Qiagen, USA), and cDNA was synthesized using Superscript IV VILO Master Mix with ezDNase (Invitrogen, USA). From the cDNA generated, the complete CDS of the gene without the stop codon was amplified using GXL Primestar (Takara, Japan) following the manufacturer’s protocol. The PCR product was directly purified after confirmation of a single band on a 1% agarose gel by using the FastGene Gel/PCR Extraction Kit (Nippon Genetic, Japan). Purified fragments were inserted into the pENTR-d-TOPO vector (Invitrogen, USA) following the manufacturer’s protocol. The plasmid was cloned into the DH5α strain (Toyobo, Japan) and sequenced to confirm the gene sequence. Gateway reaction with the plasmid pGWB442 (Nakagawa et al., 2007) was performed to fuse the YFP sequence to the S*lDET1* sequence in the N terminus using a Gateway LR Clonase Enzyme Mix Kit (Invitrogen, USA), and the product was then cloned into the DH5α strain (Toyobo, Japan). The sequence was confirmed a second time to prevent any insertion mutations.

Correctly selected plasmids were inserted into the *Agrobacterium tumefaciens* GV2260 strain, and then transient transformations of 3-week-old tobacco leaves (*Nicotiana tabacum*) were performed. Bacterial culture containing our YFP-fused protein and a pBICp19 plasmid (Takeda et al., 2002) was them resuspended in a solution of 10 mM of MES-MgSO_4_ adjusted at pH5.6 and injection was performed on 4-week-old tobacco plants that were then incubated for 3 days. Nuclei were stained by infiltration of a solution of 1 mM DAPI (Nacalai Tesque, Japan) with incubation for 30 min in the dark before observation. Infected leaves were cut into small pieces containing the infected area and placed between glass slides with the abaxial side facing the objective. YFP and DAPI fluorescence signals were visualized under a confocal microscope (Zeiss LSM700, Carl Zeiss, Germany) using appropriate filters and automatic settings provided by the maker software.

### Statistical Analysis

Statistical analysis of the photomorphogenesis results was performed by an independent-samples T test and Student–Newman–Keuls test with a significant difference cutoff of *p* < 0.05. All statistical analyses were performed using the R environment^2^ The following packages were used for the statistical analysis: dplyr (Wickham et al., 2020),^3^ Rmisc (Hope, 2013),^4^ FSA (Ogle et al., 2020),^5^ and agricolae (De Mendiburu, 2020).^6^

Images of plant and fruit development, photomorphogenesis, carotenoid accumulation, and sugar and protein contents were constructed by using the package *ggplot2* version 3.3.2 (Wickham, 2016).^7^

## Results

### Mutant Selection for *SlDDB1*, *SlDET1*, and *SlCYC-B*

**Figure 1:**
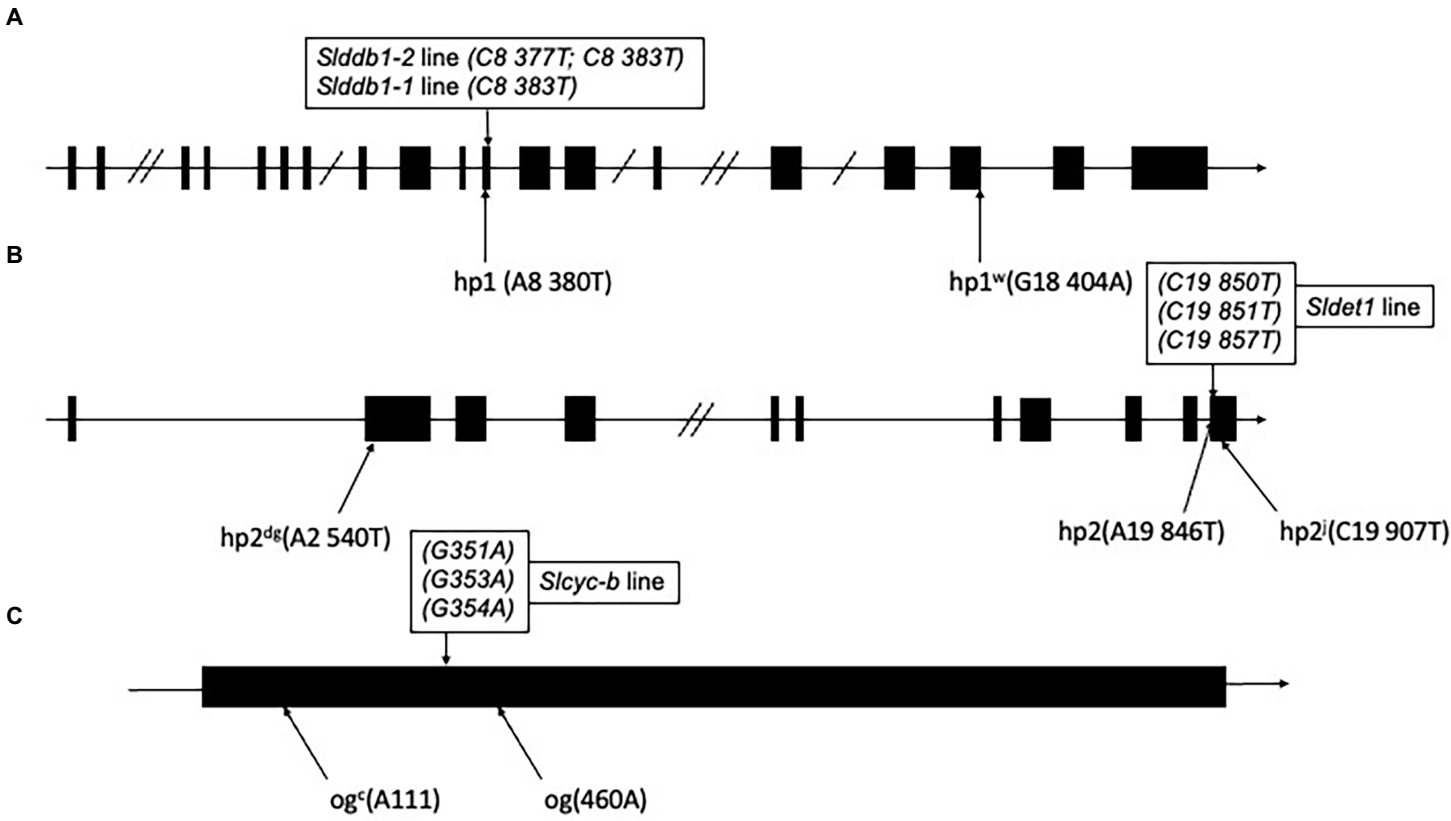
Schematic representation of individual alleles generated in Slddb1, Sldet1 and Slcyc-b. **(A)** Slddb1, **(B)** Sldet1 and **(C)** Slcyc-b showing the previously described mutations and their corresponding annotations and positions compared to the first nucleotide of the start codon in the gene sequence. Black boxes represent exon regions, and lines represent introns or UTR regions. Double slashes in introns represent a reduction in the intron size. The box above each gene represents the mutated site by Target-AID with the corresponding nucleotide position in the gene relative to the start codon.

Point mutations in *SlDDB1* (Lieberman et al., 2004; Liu et al., 2004), *SlDET1* (Mustilli et al., 1999) and *SlCYC-B* (Ronen et al., 2000), named *hp1*, *hp2*, and *og,* respectively, result in tomato lines presenting a high accumulation of lycopene. Mutations previously generated by Target-AID (Hunziker et al., 2020) were located in the vicinity of the original mutation described (Figure 1). For *SlDDB1*, the principal substitution was the cytidine located at position 8,383, which resulted in the expected substitution of Ala with Pro. This allele were hereafter called *Slddb1-1*. In the *SlDDB1* double substitution line, including the C8,383T substitution, an additional cytidine was replaced by a thymine at position 8,377 and resulted in the substitution of Asp with His. This allele was named *Slddb1-2*. Biallelic lines carrying *Slddb1-1/Slddb1-2* were included in the analysis to determine the dominance of each allele and the potential impact of improving carotenoid accumulation in biallelic plants. For *SlDET1*, mutations in our target region in exon 11 were expected to produce a nonviable NLS domain, resulting in a loss of function and an *hp* phenotype. This allele was called *Sldet1*. For *SlCYC-B*, the cytidine substitution in our target site creates a stop codon and results in a truncated protein and was renamed *Slcyc-b*. The new alleles of the gene are expected to represent a potential combination of mutated *SlDDB1* and *SlDET1*, thus inducing a complementary improvement in lycopene accumulation; however, no double mutants have been deeply analyzed in the past. Three multi-target null segregant lines (#16-3_3, #43-2_2 and #43-2_6’) were isolated and found to carry the same mutation patterns in *SlDET1* and *SlCYC-B*. Analyses were performed for the T_3_ generation to fix each mutation and prevent biallelism *Slddb1-1/Slddb1-2* within #43-2_2. The mutation pattern of each gene is summarized in Figure 1.

**Figure 2:**
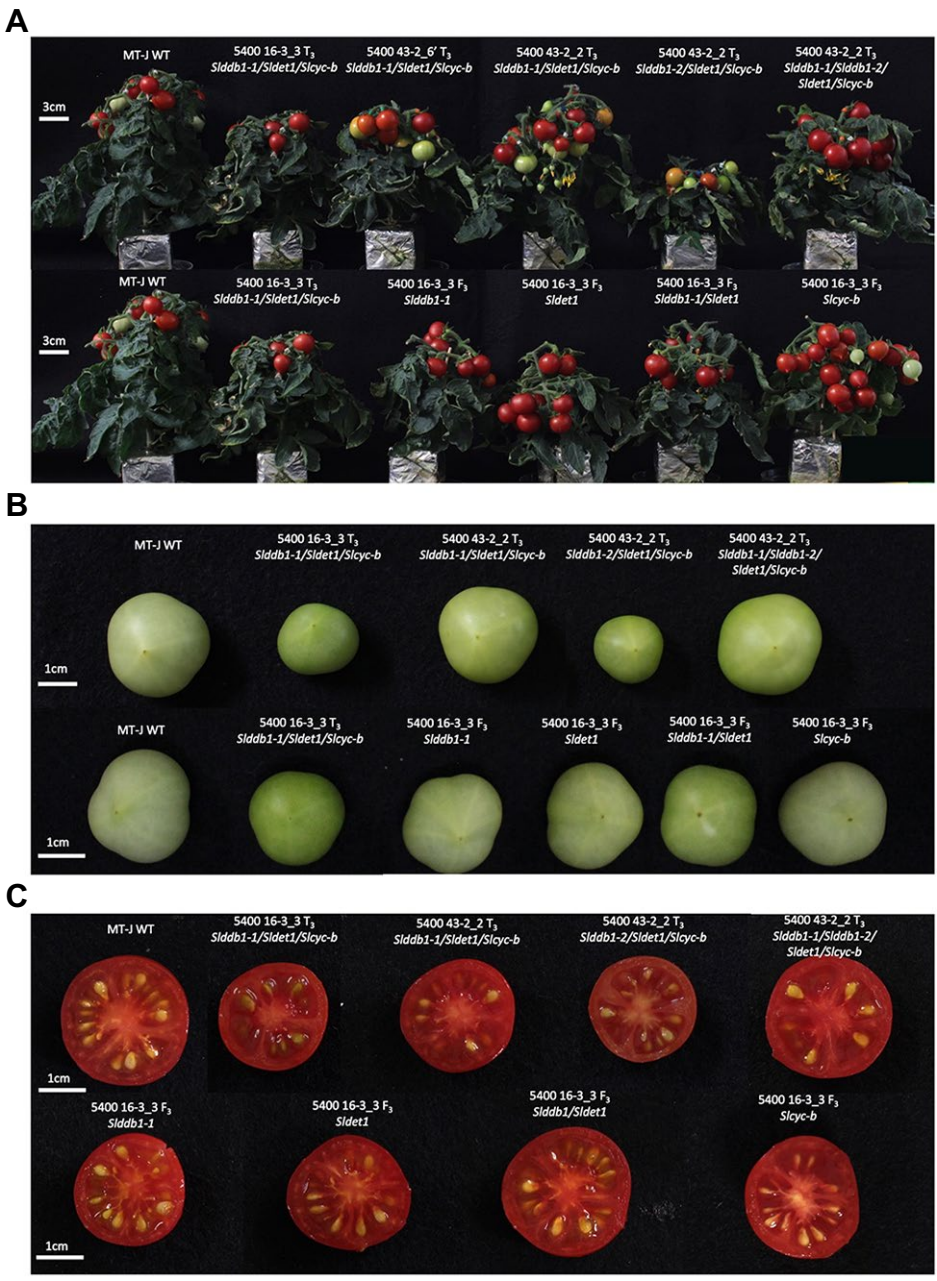
Plant and fruit phenotypes of the segregating generation. **(A)** Six-week-old plants in the T_3_ generation (upper) and BC_1_F_3_ generation (lower). **(B)** Fruit color at the green mature stage (28 days post-anthesis) in the T_3_ generation (upper) and BC_1_F_3_ generation (lower). **(C)** Fruit color at the red ripe stage (12 days post-break) in the T_3_ generation (upper) and BC_1_F_3_ generation (lower). MT-J, Micro-Tom Japan; WT, wild type. T, transformant generation; F_3_, third filial generation.

To analyze the effect of single gene mutation, a backcross population was created using the triple mutant line carrying *Slddb1-1*, as *Slddb1-2* tends to result in much more severe dwarfism, fewer fruits, and more issues with crossing and obtaining seeds (Figure 2A). The BC_1_F_3_ population was used to confirm the homozygous state of each gene.

### Plant Development of Lines With Multiple Mutations

**Figure 3:**
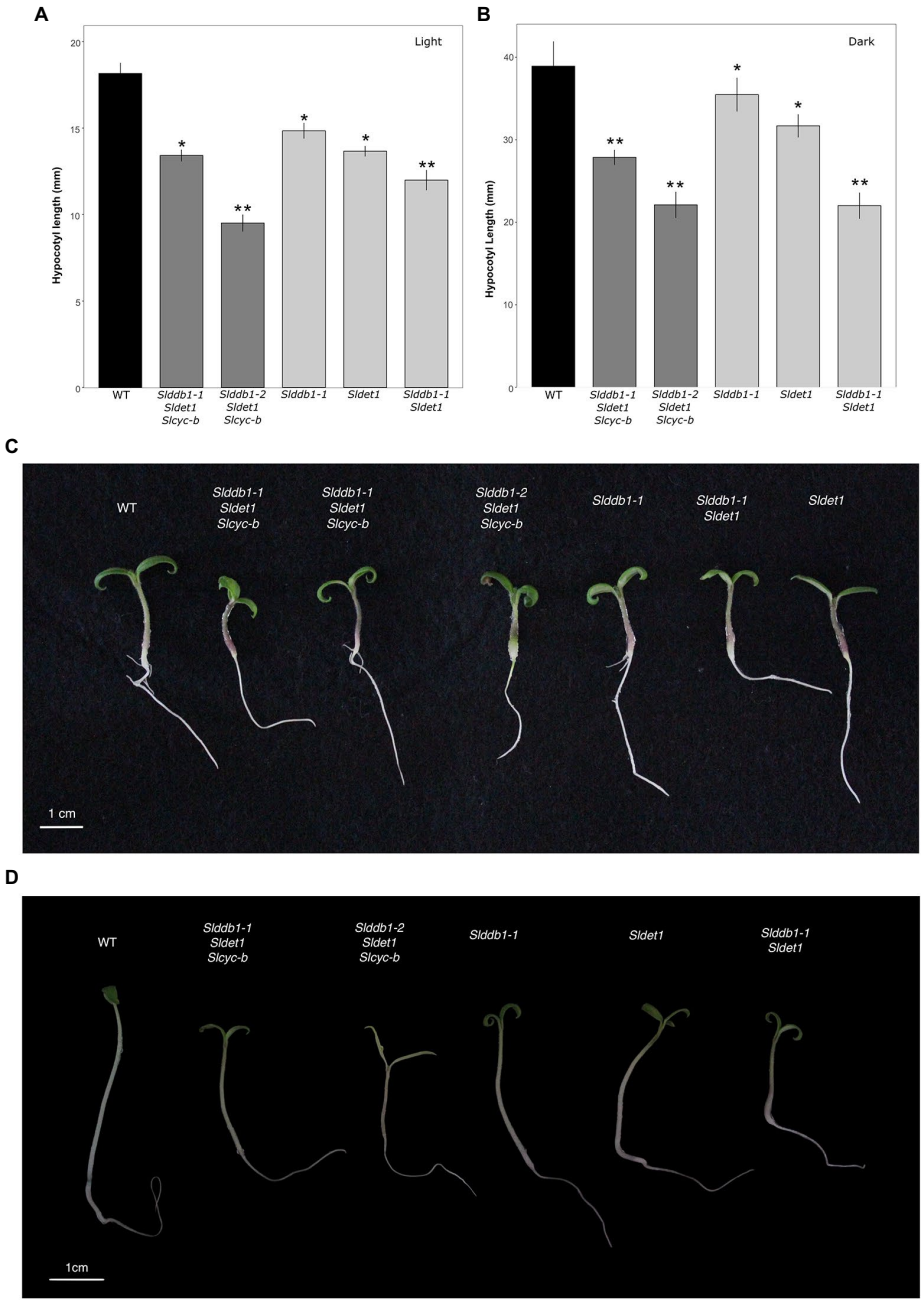
Photomorphogenesis in segregating T_3_ and BC_1_F_3_ generations. Seedling development of 7-day-old germinated seeds in the T_3_ and BC_1_F_3_ generations under light or dark conditions, as indicated. **(A,B)** Hypocotyl length measurements in the T_3_ and BC_1_F_3_ generations under light and dark conditions, respectively. Black indicates WT, dark gray indicates T_3_ triple mutant lines, and light gray indicates BC_1_F_3_ segregants. **(C)** Photography of 1 week-old germinated plantlet under light condition. White bar indicates 1 cm. **(D)** Photography of 1 week-old germinated plantlet under dark condition. White bar indicates 1 cm. Error bars indicate SE, and ^*^ and ^**^ indicate significant differences from WT and the single mutant, respectively, at 0.05 based on a *t*-test.

**Figure 4:**
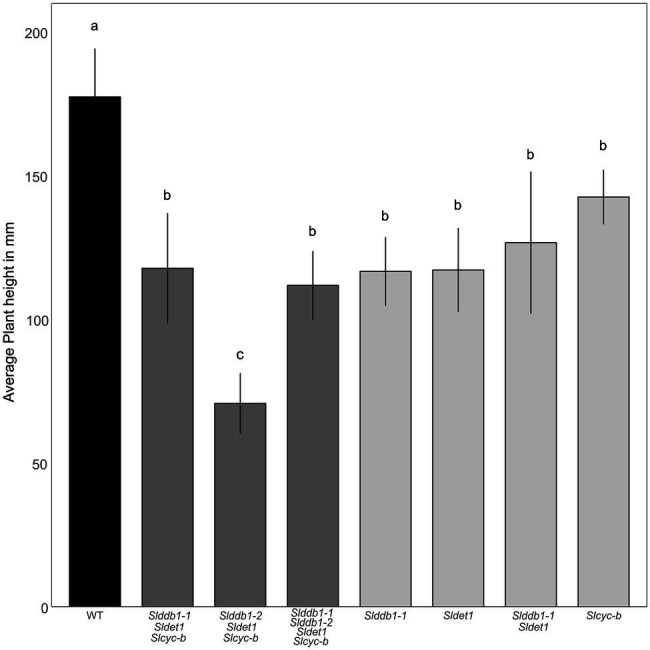
Plant development in segregating T_3_ and BC_1_F_3_ generations. Plant size was measured with 3-month-old plants from the cotyledon to the top of the plant canopy. Black indicates WT, dark gray indicates T_3_ triple mutant lines, and light gray indicates BC_1_F_3_ segregants. Error bars indicate SD, and letters and upper bars indicate groups significantly different at *p* = 0.05 according to the SNK test.

All multiple-target lines showed a typical *hp* mutant phenotype involving photomorphogenesis effects, dwarfism, dark leaves, and dark green fruits (Figure 2). As reported previously (Liu et al., 2004; Hunziker et al., 2020), we noticed a significant decrease in the hypocotyl size between the WT and *SlDDB1* and *SlDET1* mutated lines under both dark and light conditions (Figure 3). We observed the additional effect on hypocotyl growth of both alleles *Slddb1-1 and Sldet1*, as reported (Liu et al., 2004), with smaller *Slddb1-1/Sldet1* or *Slddb1-1/Sldet1*/*Slcyc-b* under light or dark conditions compared to single mutant and the WT (Figure 3). We also investigated the additional impact of each mutation on plant growth. After removing lateral shoots, we observed a significant effect of each mutation on plant size (Figures 2A, 4) compared to the WT. Interestingly, this additive phenotype from *Slddb1-1* and *Sldet1* was not observed on plant growth as it was on the hypocotyl, as each single, double or triple mutants lines did not show significant differences in size between them. *Slddb1-2/Sldet1*/*Slcyc-b* showed the stronger phenotype with the smaller size hypocotyl size, more especially under light condition (Figures 3C,D). Moreover, we could notice the development of chlorophyll within the root and wider hypocotyl in the *Slddb1-2/Sldet1*/*Slcyc-b* line compared to other mutants carrying a mutation in *Slddb1* and/or *Sldet1* under light condition (Figure 3C). Finally, the dark flower phenotype resulting from a mutation in *SlCYC-B* originally described in the *og* mutant (Ronen et al., 2000) was not observed in any mutated lines, as already described for Micro-Tom lines (Sestari et al., 2014; Hunziker et al., 2020; Supplementary Figure S1).

### Characterization of the Impact of Mutation on Fruit Development

**Figure 5:**
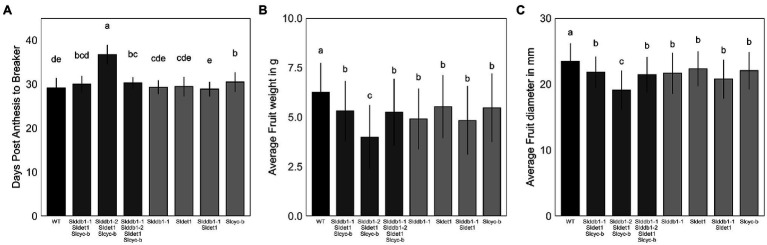
Fruit development in segregating T_3_ and BC_1_F_3_ generations. Fruit development was measured at the post-mature green stage (28 DPA). **(A)** Fruit development from the anthesis stage (0 DPA) to the breaker stage, corresponding to the end of fruit growth and the beginning of maturation. **(B,C)** Fruit weight in g and equatorial size in mm measured at the post-break stage. Error bars indicate SD, and letters and upper bars indicate groups significantly different at *p* = 0.05 according to the SNK test.

*hp* mutants have been reported to show a delay in fruit development (Cruz et al., 2018). The effect on fruit development from the anthesis stage to the breaker stage was investigated to confirm the results of previous reports on commercial cultivars. We noticed a strong impact of homozygote *Slddb1-2* in the triple mutant line, with a delay in fruit development of approximately 5 additional days to reach the breaker stage compared to other mutant lines. Interestingly, WT lines showed a similar fruit development stagetime from the anthesis stage to the breaker stage, between 28 to 30 days, compared to the different lines carrying the *Slddb1-1* and/or *Sldet1* mutation, in contrast to previous reports (Wang et al., 2019). *Slcyc-b* showed a smaller significant delay in fruit ripening (1 day) than the WT but was still in the same range as *Slddb1-1/Slddb1-2/Sldet1*/*Slcyc-b* plants and *Slddb1-1/Sldet1*/*Slcyc-b* (Figure 5A).

Regarding fruit weight and size, we could clearly observe an impact of mutations on fruit growth. All lines showed a significant decrease in fruit size and weight compared to the WT, which was stronger in the *Slddb1-2/Sldet1*/*Slcyc-b* line (Figures 5B,C).

### Evaluation of Chlorophyll Content in the T_3_ and BC_1_F_3_ Lines

*hp* mutants are known to show a higher accumulation of chlorophyll in the green stage, resulting in darker green fruits at green stages (Davuluri et al., 2004; Torres and Andrews, 2006; Kolotilin et al., 2007; Caspi et al., 2008; Jones et al., 2012). We investigated the impact of each generated allele in individual *Slddb1-1*, *Sldet1* and *Slddb1-1/Sldet1* lines on the total chlorophyll content and chlorophyll “a” and “b” individually in mature green fruits. These mutations resulted in an increase in the accumulation of total chlorophyll in all genotypes, although *Slddb1-1* did not show a significant increase compared to the WT (Table 1). We observed a significantly higher accumulation in the *Sldet1*, as observed in original *hp2* mutants and in the double mutant *Slddb1-1/Sldet1*, with additional accumulation compared to single mutants.

**Table 1:** Chlorophyll contents in μg/g^FW^ of segregating T_3_ and BC_1_F_3_ generations at the mature green stage and lycopene and β-carotene contents at the red ripe stage.

Plant line	WT	*Slddb1-1*	*Slddb1-2*	*Slddb1-1* *Slddb1-2*	*Slddb1-1*	*Sldet1*	*Slddb1-1* *Sldet1*	*Slcyc-b*
		*Sldet1*	*Sldet1*	*Sldet1*				
		*Slcyc-b*	*Slcyc-b*	*Slcyc-b*				
Chlorophyll ‘a’	25.3 ± 1.0 c	42.1 ± 0.9 b	76.9 ± 6.1 a	NA	28 ± 0.9 c	35.1 ± 1.0 b	39.7 ± 1.6 b	28.5 ± 1.2 c
Chlorophyll ‘b’	10.2 ± 0.4 c	16.5 ± 0.3 b	26.7 ± 2.3 a	NA	11.2 ± 0.3 c	14.2 ± 0.4 b	15.3 ± 0.6 b	11.7 ± 0.5 c
Total chlorophyll	35.5 ± 1.4 c	58.6 ± 1.2 b	103.6 ± 8.3 a	NA	39.2 ± 1.3 c	49.3 ± 1.4 b	55.1 ± 2.3 b	40.2 ± 1.7 c
Lycopene	174.2 ± 5.4 d	215.2 ± 2.7 ab	172.5 ± 8.7 d	201.8 ± 5.0 bc	192.6 ± 3.2 c	192.5 ± 2.6 c	198.5 ± 4.9 bc	215.0 ± 2.9 ab
β-Carotene	19.8 ± 0.5 c	29.9 ± 0.4 b	33.1 ± 0.9 a	28.7 ± 0.9 bc	30.0 ± 0.6 b	30.5 ± 0.5 b	28.9 ± 0.8 b	33.5 ± 0.6 a

Measurement of chlorophyll, lycopene and β*-carotene contents in the fruit pericarp by spectrophotometry at 28 DPA for chlorophyll and 12 DPB for lycopene and* β-carotene in T_3_ and BC_1_F_3_ segregants, respectively. Values are mean SE and expressed in μg/g^FW^ Letters and upper bars indicate groups significantly different at p = 0.05 according to the SNK test. NA, data not applicable.

Interestingly, *Slcyc-b* also showed an increase in the total chlorophyll content that was comparable to the content of the *Slddb1-1*, but this increase was not significantly different from that in the WT. These observations were correlated with the visual observations of the fruit from each line, with these fruits showing clear color difference for *Slddb1-1* and *Slcyc-b* mutants compared to *Sldet1* and *Slddb1-1/Sldet1* mutants (Figure 2B). Finally, triple mutant lines showed the most significant increase compared to all other genotypes, with a pattern of accumulation of chlorophyll that was higher than all other lines except the *Slddb1-1/Sldet1*, which also had a noticeable effect on fruit color (Figure 2B; Table 1). *Slddb1-2/Sldet1*/*Slcyc-b* showed the strongest phenotype, with the darkest fruit containing the highest amounts of chlorophyll (Table 1). However, the individual impact of the double-substituted allele *Slddb1-2* could not be determined due to the difficulty of producing viable plants and fruits.

### Evaluation of Carotenoid Accumulation in the T_3_ and BC_1_F_3_ Lines

A BC_1_F_3_ population of #16-3_3 from our previous study (Hunziker et al., 2020) was generated to segregate each mutation and analyze its impact on fruit development and carotenoid accumulation. Five fruits per plant were harvested at 12 days post-break (DPB). Only the pericarp was kept preventing bias resulting from the whiter columella color in the WT. The lycopene and β-carotene contents were measured by spectrophotometry, as lycopene is a major carotenoid in tomato fruits. As each of the null segregant lines #16-3_3 T_1_, #43-2_6’ T_1_ and #43-2_2 T_1_ presented a different mutation pattern in *SlDDB1*, #43-2_2 being biallelic *Slddb1-1/Slddb1-2* (Hunziker et al., 2020), differences in the carotenoid contents and accumulation were expected; thus, T_3_ lines were analyzed to compare each genotype of #43-2_2.

Lines #16-3_3, #43-2_6’ and #43-2_2 T_3_ *Slddb1-1/Sldet1*/*Slcyc-b* were merged together since no significant difference could be observed. The results of the analysis of the T_3_ lines showed an increase in lycopene of almost 30% in *Slddb1-1/Sldet1*/*Slcyc-b* compared to the WT, whereas single mutants *Slddb1-1*, *Sldet1* and *Slddb1-1/Sldet1* showed an increase of approximately 12% (Table 1). Similar to the effect on plant growth, a combined effect of *Slddb1-1* and *Sldet1* mutations on lycopene accumulation could be observed, with a gradual increase found for individual *Slddb1-1*, *Sldet1,* to *Slddb1-1/Sldet1* and *Slddb1-1/Sldet1*/*Slcyc-b*. Interestingly, the *Slcyc-b* mutant showed higher accumulation within BC_1_F_3_ lines, which was close to that of the triple mutant, without showing the dark phenotype on the columella and placenta (Figure 2C; Table 1). The *Slddb1-2/Sldet1*/*Slcyc-b* line showed a significant decrease in lycopene and β-carotene accumulation compared to the WT, even though fruits were harvested on the same timescale, as the mutant line showed potentially delayed fruit ripening, similar to what was observed for fruit development (Figure 2C; Table 1). Confirmation of the hypothesis could not be performed on later generations due to the limited number of seeds and fruits produced.

The accumulation of β-carotene was expected to be reduced in mutant lines compared to WT, resulting in an expected disruption of the CYC-B enzyme. Interestingly, we observed an increase in β-carotene of more than 50% in the T_3_ generation, with even more in *Slcyc-b* and *Slddb1-2/Sldet1*/*Slcyc-b* lines (Table 1).

Such observations of a higher accumulation of lycopene and β-carotene in triple-substituted lines and *Slddb1-1*, *Sldet1* and *Slddb1-1/Sldet1* are similar to what was observed in a previous report on triple-substituted T_1_ lines (Hunziker et al., 2020). These results correlated with those for fruit color at the green stage. In *Slddb1-1*, *Sldet1, Slddb1-1/Sldet1* and triple mutated lines, a darker color was observed (Figure 2B), which resulted from a higher content of chlorophyll compared to the WT line (Table 1) and higher total carotenoids at the red stage (Table 1), as reported for the *hp* tomato mutant (Cookson et al., 2003; Davuluri et al., 2004; Torres and Andrews, 2006).

### Evaluation of Sugar and Protein Contents in the T_3_ and BC_1_F_3_ Lines

**Figure 6:**
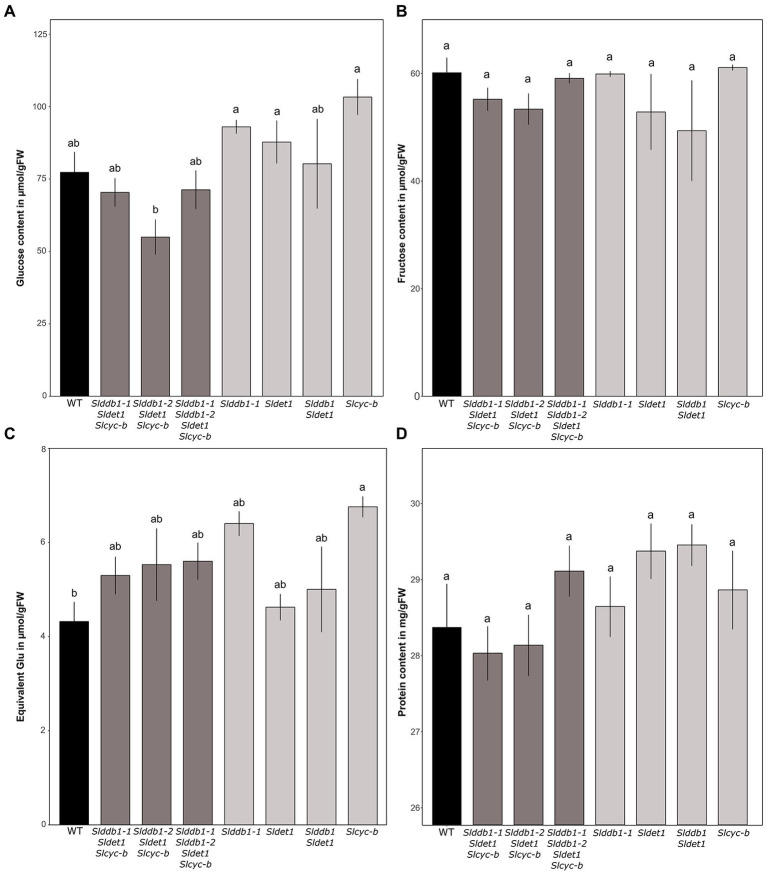
Sugar contents in μmol.g^−1^FW and protein contents of segregating T_3_ and BC_1_F_3_ generations at the red ripe stage. **(A–C)** Measurement of glucose, fructose and sucrose contents in the fruit pericarp by spectrophotometry at 12 DPB in T_3_ and BC_1_F_3_ segregants, respectively. Concentrations are expressed in μmol.g^−1^FW for glucose and fructose and equivalent glucose in μmol.g^−1^FW for sucrose. **(D)** Protein content measured at 12 DPB in T_3_ and BC_1_F_3_ segregants. The content is expressed in mg/gFW. Black indicates WT, dark gray indicates T_3_ triple mutant lines, and light gray indicates BC_1_F_3_ segregants. Error bars indicate SD, and letters and upper bars indicate groups significantly different at *p* = 0.05 according to the SNK test.

An investigation of sugar accumulation was conducted on ripe fruits to determine whether the mutations generated impacted the taste of the fruit. Previous analysis on cultivars showed a variable impact of the *hp1* mutation on the sugar content (Yen et al., 1997; Enfissi et al., 2010; Pal et al., 2019). Glucose tended to show higher accumulation in our single mutant lines (Figure 6A). The increase in glucose in the BC_1_F_3_ lines ranged from 20% in *Slddb1-2*, *Sldet1* and the double mutant *Slddb1-1/Sldet1* to 25% in *Slcyc-b*. For triple mutant transformant lines, we could not observe a significant difference between *Slddb1-1/Sldet1*/*Slcyc-b* line and *Slddb1-1/Slddb1-2/Sldet1*/*Slcyc-b*, whereas *Slddb1-2/Sldet1*/*Slcyc-b* showed a significant decrease of approximately 20%. ANOVAs of the fructose analyses appeared to show a nonsignificant difference between all mutated lines and the WT (Figure 6B), thus all lines were not considered to be different. Finally, compared to the WT, we noticed a tendency for a higher accumulation of sucrose in all triple mutant lines and backcrossed lines (Figure 6C), with an increase of almost 50% in *Slddb1-1and Slcyc-b*.

Total protein content appears to not be significantly altered, as all lines showed the same amount of protein in the pericarp (Figure 6D).

### Evaluation of the Loss of Function of the NLS Domain in *SlDET1*

**Figure 7:**
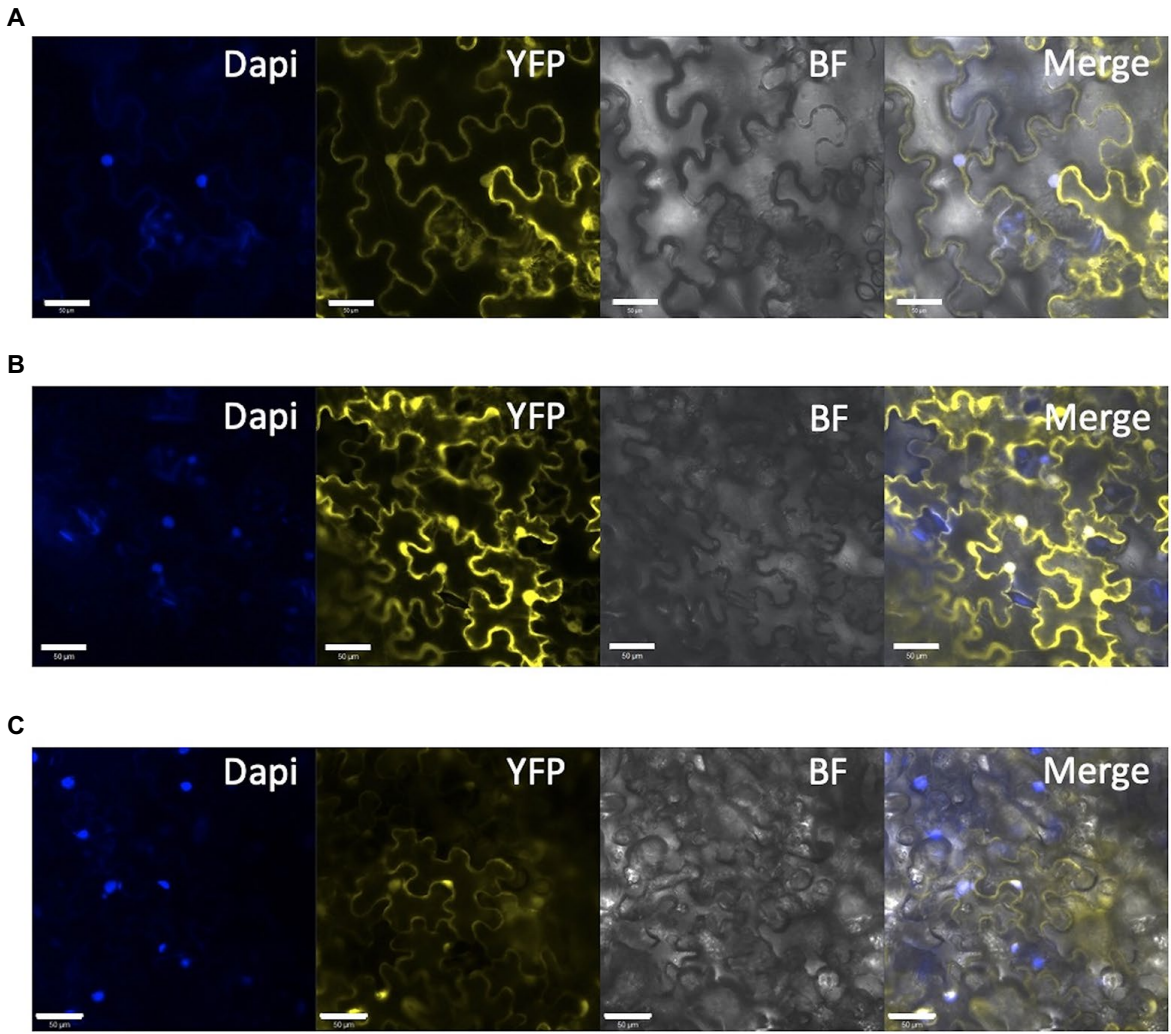
Subcellular localization of mutated SlDET1 generated by Target-AID. **(A,B)** Confocal microscopy image of YFP-SlDET1 WT or mutated fusion protein in yellow; DAPI nucleus staining is shown in blue; BF represents the bright field image; and Merge show the merged image of the infected tobacco leaf. **(C)** 35S::YFP controlling transient expression in tobacco leaves. We noticed a strong protein signal in the cytoplasm and nucleus of the cell in both forms. White bars represent scale of 50 μm.

As reported previously (Mustilli et al., 1999), our target on SlDET1 is located in the NLS domain of the protein. The *hp2* allele shows a nontotal loss of function, resulting in approximately 10% of the mRNA transcript being correctly spliced (Mustilli et al., 1999). Our previous screening of the T_1_ and T_2_ generations could not isolate any homozygous indels in the gene in the triple mutant, suggesting a potential functional NLS domain in our mutated protein (Hunziker et al., 2020). To confirm the loss of function of the localization signal of the protein in the nucleus, protein fusion of YFP to the N terminus (YFP-*Sl*DET1) of both the WT and mutated versions of the protein was performed, as fusion in the C terminus (*Sl*DET1-YFP) results in reduced NLS function of the protein, as previously reported (Schroeder et al., 2002). Transient expression in tobacco leaves by Agrobacterium infiltration and analysis of fluorescent signals under confocal microscopy revealed subcellular localization of the WT signal in the nucleus, as confirmed by DAPI staining, and in the cytoplasm (Figure 7A). Regarding the loss of function of the NLS protein, we observed a similar localization of mutated proteins in the cytoplasm and nucleus (Figure 7B). Such observations are similar to the WT positive control 35S::*SlDET1*-*GFP* reported by Thakare et al. (2020) but contradict the results reported by Mustilli et al. (1999) and Schroeder et al. (2002).

## Discussion

In this study, we characterized the impact of newly identified weak alleles generated by Target-AID and their additive effect on carotenoid accumulation. Two of the selected target genes and mutated alleles characterized in tomato were described as strongly impacting hypocotyl growth (Mustilli et al., 1999; Levin et al., 2003; Lieberman et al., 2004; Azari et al., 2010) and fruit development (Wang et al., 2019). Our study could confirm the impact of newly created alleles on photomorphogenesis, but with lower impact under darkness condition (Figure 3). Target-AID is an efficient breeding tool for inducing multiple-gene cytidine substitutions in tomato, as reported in our previous study (Hunziker et al., 2020). The present results confirm that this method helps produce weak mutated alleles in genes of interest that have reduced impacts on plant growth. Previous reports in *Arabidopsis* and tomato showed significant deleterious effects of the combination of *DDB1* and *DET1* mutated alleles, such as extreme dwarfism and sterility of the plant (Schroeder et al., 2002; Liu et al., 2004). However, Target-AID base-editing technology can generate double/triple mutants without extreme dwarfism or sterility, as observed in the *Slddb1-1/Sldet1*/*Slcyc-b* or *Slddb1-1/Sldet1* lines used here (Figure 2). However, the choice of Micro-Tom, an extreme dwarf brassinosteroid deficient tomato cultivar., may attenuate the pleiotropic effects of *Slddb1* and *Sldet1* on plant growth and fruit development. A similar observation to the tomato brassinosteroid deficient *dpy* and *cu-3* mutants (Koka et al., 2000) was done on photomorphogenesis, with a smaller hypocotyl under dark condition (Figure 2C).

Substitution in complementation of knockout of candidate genes remain important in breeding, with the use of new breeding technologies. No truncated mRNA of *SlDDB1* has been reported previously in tomato, and only *SlDET1* truncated at the C terminus has been described; however, a small amount of functional protein remained, confirming the necessity of both nontruncated proteins in plant development. In our previous study (Hunziker et al., 2020), the absence of homozygous plants for an indel in *SlDDB1* or *SlDET1* was explained by the necessity for a small amount of both functional proteins, as no T_2_ homozygous indel lines with both genes truncated simultaneously could be obtained (Hunziker et al., 2020). Reports on splicing variants in *Arabidopsis* showed a stronger effect of *AtDET1* truncation on plant growth, resulting in extreme dwarfism (Pepper and Chory, 1997; Schroeder et al., 2002).

For *SlDET1*, the original *hp2* mutation led to alternative splicing, resulting in a deletion of three amino acids in exon 11 and a truncated NLS and C terminus of the protein (Mustilli et al., 1999). However, this mutation was described as an incomplete knockout mutation, with approximately 10% of the mRNA being correctly spliced. The same observation could be made for Arabidopsis *AtDET1,* which had correct RNA splicing in less than 1 to 2% of proteins (Pepper and Chory, 1997), suggesting the importance of the presence of nontruncated proteins. This allele retains the full-size protein, with only a double amino acid substitution in the NLS domain, which suggests the importance of the C terminus in the DET1 protein. Based on the subcellular localization results of the *Sl*DET1 protein (Figure 7B), successful loss of cellular localization could not be achieved. The WT protein was localized not only in the nucleus, as previously reported in tomato (Mustilli et al., 1999) and *A. thaliana* (Schroeder et al., 2002), but also in the cytoplasm. However, images in both previous publications in tomato and *Arabidopsis* are slightly ambiguous, as precautious observation does not suggests a total nucleus specificity. A similar observation of cytoplasmic and nuclear localization was found in a more recent publication (Thakare et al., 2020) with a DET1-GFP, which had potential interference from the GFP in the C terminus, as observed in *Arabidopsis* (Schroeder et al., 2002). The presence of the mutated protein in the nucleus was associated with an impact on the plant phenotype and could result in a change in the confirmation of the protein. The amino acid substitutions P479F and A481V may result in an alteration in the interaction with other proteins of the CDD complex, similar to the double mutant *Slddb1-1/Sldet1,* which showed a combined effect from both mutations. The impact of the mutation on morphogenesis and plant growth was similar to what was already observed with the *hp2* allele in tomato and *AtDET1* mutants, with small seedlings of edited plants showing a reduced effect under dark growth conditions (Figure 4; Pepper and Chory, 1997; Mustilli et al., 1999; Levin et al., 2003; Liu et al., 2004; Carvalho et al., 2011).

Two alleles of *SlDDB1* could be isolated in this study. As *Slddb1-1* allele resulted in a weaker effect of the mutation in triple mutant background, this allele was subjected to further analysis due to the difficulty of isolating and maintaining triple mutant lines carrying *Slddb1-2*. The C8,383T mutation was located 4 bp downstream of the original *hp1* mutation, resulting in the substitution of an alanine with a threonine in a less strongly conserved domain that for the preceding asparagine to tyrosine substitution in *hp1* (Lieberman et al., 2004; Hunziker et al., 2020). This location might explain why the allele was weaker compared to the original *hp1* introgressed into Micro-Tom (Carvalho et al., 2011). The biallelic condition of the gene did not induce a significant change in plant size in the T_3_ generation neither in carotenoid accumulation when analyzed by spectrophotometry (Table 1), in contrast to the #43-2_2 T_1_ generation that was analyzed by HPLC (Hunziker et al., 2020), suggesting a possible variation or bias in the sampling between the two generations. Moreover, it concludes on the dominance of Slddb*1-1* on *Slddb1-2*. The same impact of *Slddb1-2* in triple mutant genotype on photomorphogenesis could be observed in the shorter hypocotyls when compared to *Slddb1-1* triple mutant lines under both light and dark conditions (Figure 3). Delays in flower and fruit development (Figures 4, 5), higher accumulation of chlorophyll and β-carotene, and lower lycopene and sugar accumulation could be observed (Table 1), suggesting a delay in ripening, as observed previously for *hp1* (Wang et al., 2019). Such combined results suggest that *Slddb1-2*, as a strong deleterious allele, is not suitable for improving agronomic traits; when in combination with our *Sldet1*, in the resulting plants were difficult to handle.

The truncated protein *SlCYC-B* produced a noneffective protein, resulting in the accumulation of lycopene in the single mutant. Both original *og* and *og^c^* mutants have been reported to contain a single nucleotide insertion resulting in a frameshift, which results in the nonlethal knockout of the gene. The mutants with a nonfunctional protein were expected to show a darker orange flower phenotype compared to the WT, as described for *og* mutants (Ronen et al., 2000). Interestingly, as reported in the T_1_ triple mutant (Hunziker et al., 2020), the T_3_ segregating generation and single mutant BC_1_F_3_ generation used in this study did not present such a phenotype of tawny orange flowers. The same observation was found in NIL Micro-Tom mutants (Sestari et al., 2014), suggesting an impact of environmental conditions or Micro-Tom genetic background on flower color. A similar observation regarding the flower color was also seen in our laboratory in commercial T_0_ lines where conventional Cas9 was used to induce a frameshift upstream of our target in the Target-AID system, suggesting a potential effect of the cultivar on the flower color phenotype (data not shown). The *Sl*CYC-B protein functions as a chromoplastic lycopene-β-cyclase, which catabolizes lycopene to β-carotene, suggesting a decrease in β-carotene concentration in pigmented tissue such as in fruits, as reported in M82 cultivar (Ronen et al., 2000). In our loss-of-function *Slcyc-b* lines, we did not observe such a decrease in the β-carotene content but indeed an increase in this content in the *Slcyc-b* single mutant line. The lack of a decrease in β-carotene in *Sl*CYC-B-defective lines has already been noted in the Micro-Tom background. A previous report also noted a lack of accumulation of lycopene, suggesting a weak isolated allelic version of the gene (Sestari et al., 2014). An increase in the lycopene content was found for the single *Slcyc-b* mutant, which was similar to that for *Slddb1-1/Sldet1*/*Slcyc-b*, suggesting a disruption of its function. A potential hypothesis regarding these color differences and their relationship to *Slddb1-1/Sldet1*/*Slcyc-b* and accumulation of β-carotene could be that there is an equilibrium between the amount of lycopene inducing a darker red color of the fruit and β-carotene inducing a clearer orange color. No orthologous gene of CYC-B has been reported in tomato, and the other lycopene-β-cyclase, LCY, has been described as a chloroplast protein. A recent study in a *de novo* domestication of tomato using a conventional Cas9 system to create a truncated protein of *Sl*CYC-B in *Solanum pimpinellifolium,* similar to our approach with Target-AID, resulted in a higher accumulation of lycopene in flowers and fruits of edited lines without negatively affecting the content of β-carotene, with a potential increase, similar to our observation for the fruit β-carotene content (Zsögön et al., 2018). These results suggest the potential compensation of the lack of activity from *Sl*CYC-B by the chloroplastic lycopene-β-cyclase LCY.

Regarding fruit quality, different observations could be made in our single and multiple mutant lines compared to previous reports. The *hp1* mutant shows a significant decrease in glucose and fructose with a higher increase in sucrose in red ripe fruits of Ailsa Craig (Yen et al., 1997) according to Yen et al. (1997) and a nonsignificant change in the accumulation of both sucrose and glucose associated with a decrease in fructose content was observed in several different *hp2* mutants within different cultivated backgrounds (Enfissi et al., 2010) according to Enfissi et al. (2010). However, another recent observation by Pal et al. (2019) was in contradiction with these previous results, which noticed an increase in the glucose and fructose content associated with an increase in the sucrose content for all independent mutants *hp1*, *hp2* and *og^c^*. In the case of our single mutants *Slddb1-1, Sldet1* and *Slcyc-b* in Micro-Tom, we observed a similar result by Pal et al. (2019) concerning the glucose and sucrose content, with an increase in their accumulation (Figure 6A). However, observations on fructose were different. Pal et al. (2019) reported an increase in fructose for all mutants, whereas we observed a nonsignificant decrease in *Slddb1-1* and *Sldet1*, similar to what was observed by Yen et al. (1997) and Enfissi et al. (2010) in *hp1* and *hp2*, respectively (Figure 6B). For *Slcyc-b* mutant, similar observations by Pal et al. (2019) with the *og^c^* mutant of an increase of the glucose and sucrose content were made (Figures 6A,C). However, we did not observe any changes in fructose contents (Figure 6B) compared to the increase in the *og^c^* mutant noticed by Pal et al. (2019). The decrease in glucose in *Slddb1-2/Sldet1*/*Slcyc-b* (Figure 6A) could be explained as a potential effect of the delay in ripening, as orange fruits tend to show lower glucose accumulation than red ripe fruits for both mutated genes (Pal et al., 2019). Our analysis resulted in a general increase of glucose and sucrose content in single mutant lines, whereas the fructose content remains unchanged (Figure 6), presenting a different sugar accumulation pattern compared to the previous reports. However, the impact of the Micro-Tom cultivar used should not be excluded. Interestingly, compared to the carotenoid accumulation, we could not observe a significant additional effect of the mutations on sugar contents within the *Slddb1-1/Sldet1*, *Slddb1-1/Sldet1/Slcyc-b* and *Slddb1-1/Slddb1-2/Sldet1/Slcyc-b*.

## Conclusion

In conclusion, Target-AID is an efficient tool for inducing new alleles and creating a mutant population without the necessity of screening a large mutagenized population by TILLING and performing backcrosses to remove undesirable mutations. Weaker alleles of *SlDDB1* and *SlDET1* could be created, providing the possibility of combining their effects on lycopene accumulation in edited mutants, which has not been reported until now in introgression lines. An exception was a double RNAi fruit-specific silenced line in tomato which was not further described (Liu et al., 2009). The impact of the mutation in *SlCYC-B* on carotenoid accumulation was confirmed with a single mutated line, suggesting its involvement in the higher accumulation of lycopene in triple mutant lines compared to double *SlDDB1/SlDET1* lines. Interestingly, rather than improving lycopene accumulation alone, β-carotene accumulation could be increased, suggesting some activity of lycopene-β-cyclase from its chloroplastic form.

## Data Availability Statement

The datasets presented in this study can be found in online repositories. The names of the repository/repositories and accession number(s) can be found in the article/Supplementary Material.

## Author Contributions

JH, TA, and HE wrote the main manuscript text, prepared figures, and supplemental information. JH did experiments. All authors contributed to the article and approved the submitted version.

## Funding

This work was supported by the Cabinet Office, Government of Japan, Cross-ministerial Strategic Innovation Promotion Program (SIP), “Technologies for creating next-generation agriculture, forestry, and fisheries” (Funding agency: Bio-oriented Technology Research Advancement Institution, NARO), grant number 14537460.

## Conflict of Interest

The authors declare that the research was conducted in the absence of any commercial or financial relationships that could be construed as a potential conflict of interest.

## Publisher’s Note

All claims expressed in this article are solely those of the authors and do not necessarily represent those of their affiliated organizations, or those of the publisher, the editors and the reviewers. Any product that may be evaluated in this article, or claim that may be made by its manufacturer, is not guaranteed or endorsed by the publisher.

## Footnotes

^1^ http://helix.wustl.edu/dcaps

^2^ http://www.R-project.org

^3^ https://CRAN.R-project.org/package=dplyr

^4^ https://cran.r-project.org/package=Rmisc

^5^ https://github.com/droglenc/FSA

^6^ https://cran.r-project.org/package=agricolae

^7^ https://ggplot2.tidyverse.org
