# Supplementary material for: Phenotypic Characterization of High Carotenoid Tomato Mutants Generated by the Target-AID Base-Editing Technology
Source: Front Plant Sci. 2022 Jul 7;13:848560. doi: 10.3389/fpls.2022.848560 (PMC9301137; doi:10.3389/fpls.2022.848560)
Supplement: Supplementary file 1 [file Data_Sheet_1.docx]

# **Phenotypic characterization of high carotenoid tomato mutants generated by the Target-AID base-editing technology**

**Johan Hunziker^1,2^, Keiji Nishida^2^, Akihiko Kondo^2^, Tohru Ariizumi^3,4^ Hiroshi Ezura^3,4^**

^1^ Graduate School of Life and Environmental Sciences, University of Tsukuba, Tsukuba, Japan.

^2^ Graduate School of Science, Technology and Innovation, Kobe University, Kobe, Japan.

^3^ Faculty of Life and Environmental Sciences, University of Tsukuba, Tsukuba, Japan.

^4^Tsukuba Plant Innovation Research Center, University of Tsukuba, Tsukuba, Japan

*** Correspondence:**

Hiroshi Ezura

**e-mail:** [**ezura.hiroshi.fa@u.tsukuba.ac.jp**](mailto:ezura.hiroshi.fa@u.tsukuba.ac.jp)

**tel: (81) 029-853-7263**

**Keywords: Carotenoid accumulation, base editing, high-pigment, tomato, new breeding technology.**


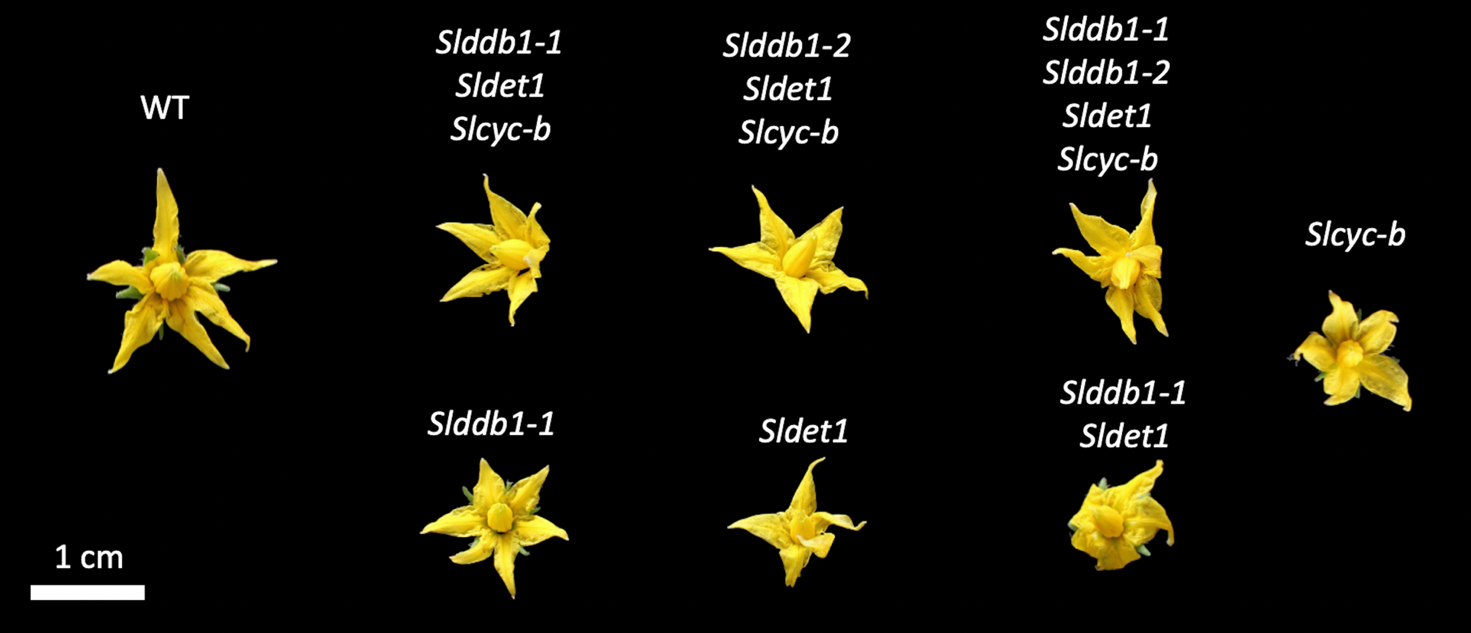


**Supplementary Figure S1. Comparison of flower color and shape of the edited lines in the T_3_ and BC_1_F_3_ generation.** Each flower was harvested at the anthesis stage from the first inflorescence on the third or fourth position on the inflorescence to prevent bias of smaller flowers resulting from lateral shoots or abnormal flower. Bar = 1cm

**Table S1. Primer sequence list used for gene cloning, sequencing, and mapping.**

| Name | Sequence | Amplified region | Used for |
| --- | --- | --- | --- |
| NPTII-F | 5'-ATGATTGAACAAGATGGATTGCAC-3' | *NPTII* | Detection of transgene |
| NPTII-R | 5'-TCAGAAGAACTCGTCAAGAAGGCG-3' | *NPTII* | Detection of transgene |
| Actin-F | 5'-GATGGATCCTCCAATCCAGACACTGTA'-3' | *Actin* | Detection of genome DNA |
| Actin-R | 5'-GTATTGTGTTGGACTCTGGTGATGGTGT'-3' | *Actin* | Detection of genome DNA |
| DET1_ON target-F1 | 5'-TCAGCCCTCTCATCCATACC-3' | *Solyc01g056340* | CAPS analysis/Sequencing |
| DET1_ON target-R1 | 5'-CGTCTTGGCACTCTATCAAGC-3' | *Solyc01g056340* | CAPS analysis/Sequencing |
| CYC_ON target-F1 | 5'-TCCACCTCCCTCCATAATTA-3' | *Solyc06g074240* | CAPS analysis/Sequencing |
| CYC_ON target-R1 | 5'-CACTCTGTTCTCAACACAAC-3' | *Solyc06g074240* | CAPS analysis/Sequencing |
| DDB1_HindIII_WT_FW | 5'-ATTGCATCAACCATATCATACCTAGACAAA-3' | *Solyc02g021650* | CAPS analysis |
| DDB1_ON target-R1 | 5'-GCCTTTCCAGATCAACAACACAGAAGTCCA-3 | *Solyc02g021650* | CAPS analysis/Sequencing |
| DET1_topo_Fw | 5’-CACCATGTTCAAAACTAACAATGTTACCG-3’ | *Solyc01g056340* | cDNA cloning |
| DET1_topo_Rv | 5’-TCGACGAAAATGGATATTTACAA-3’ | *Solyc01g056340* | cDNA cloning |
| DET1_Seq1_Fw | 5’-ATCCATCGTGCCAGAAGATT-3’ | *Solyc04g005070* | cDNA Sequence |
| DET1_Seq2_Fw | 5’-TGTCCTCACACTCAAACAACATCCA-3’ | *Solyc01g056340* | cDNA Sequence |
| DET1_Seq3_Rv | 5’-GTCAAACCTGAAGAGAGATTGGTC-3’ | *Solyc01g056340* | cDNA Sequence |
| DET1_Seq1_Rv | 5’-CAGTAGGAGGTGCATC-3’ | *Solyc01g056340* | cDNA Sequence |
| DET1_Seq2_Rv | 5’-CTGATTGAGGCAGACTATGATGA-3’ | *Solyc01g056340* | cDNA Sequence |
